# Cyclization-blocked proguanil as a strategy to improve the antimalarial activity of atovaquone

**DOI:** 10.1038/s42003-019-0397-3

**Published:** 2019-05-03

**Authors:** Tina S. Skinner-Adams, Gillian M. Fisher, Andrew G. Riches, Oliver E. Hutt, Karen E. Jarvis, Tony Wilson, Mark von Itzstein, Pradeep Chopra, Yevgeniya Antonova-Koch, Stephan Meister, Elizabeth A. Winzeler, Mary Clarke, David A. Fidock, Jeremy N. Burrows, John H. Ryan, Katherine T. Andrews

**Affiliations:** 1 Griffith Institute for Drug Discovery, Griffith University, Nathan, QLD 4111 Australia; 2 Commonwealth Scientific and Industrial Research Organization, Biomedical Manufacturing, Clayton, VIC 3168 Australia; 3 Institute for Glycomics, Griffith University Gold Coast Campus, Gold Coast, QLD 4222 Australia; 4 School of Medicine, University of California, San Diego, La Jolla, CA 92093 USA; 5 Department of Microbiology and Immunology, and Division of Infectious Diseases, Department of Medicine, Columbia University Medical Center, New York, NY 10032 USA; 6 Medicines for Malaria Venture (MMV), Route de Pré Bois 20, Geneva, 1215 Switzerland; 7 Present Address: California Institute for Biomedical Research (Calibr), La Jolla, CA 92037 USA; 8 Present Address: Beckman Coulter Life Sciences in Indianapolis, Indianapolis, IN 46268 USA

**Keywords:** Drug discovery, Diseases

## Abstract

Atovaquone-proguanil (Malarone®) is used for malaria prophylaxis and treatment. While the cytochrome bc1-inhibitor atovaquone has potent activity, proguanil’s action is attributed to its cyclization-metabolite, cycloguanil. Evidence suggests that proguanil has limited intrinsic activity, associated with mitochondrial-function. Here we demonstrate that proguanil, and cyclization-blocked analogue tBuPG, have potent, but slow-acting, in vitro anti-plasmodial activity. Activity is folate-metabolism and isoprenoid biosynthesis-independent. In yeast dihydroorotate dehydrogenase-expressing parasites, proguanil and tBuPG slow-action remains, while bc1-inhibitor activity switches from comparatively fast to slow-acting. Like proguanil, tBuPG has activity against *P. berghei* liver-stage parasites. Both analogues act synergistically with bc1-inhibitors against blood-stages in vitro, however cycloguanil antagonizes activity. Together, these data suggest that proguanil is a potent slow-acting anti-plasmodial agent, that bc1 is essential to parasite survival independent of dihydroorotate dehydrogenase-activity, that Malarone® is a triple-drug combination that includes antagonistic partners and that a cyclization-blocked proguanil may be a superior combination partner for bc1-inhibitors in vivo.

## Abstract (web summary)

Tina Skinner-Adams et al. show that proguanil and the cyclization-blocked analogue, tBuPG, have potent, anti-plasmodial activity in vitro. This study suggests that a cyclization-blocked proguanil may be a superior combination partner for bc1-inhibitors to suppress the survival of malarial parasites.

## Introduction

The arylbiguanide proguanil (Paludrine®) was introduced for malaria treatment in the 1940’s, however resistance to the monotherapy was rapidly detected^1^. The potent fast-acting activity of proguanil is attributed to the dihydrofolate reductase inhibitor cycloguanil (the product of liver cytochrome P450 (CYP2C19) metabolism^2^) and resistance has been shown to be mediated by dihydrofolate reductase mutations^3^. To overcome clinical parasite resistance, and following the finding that proguanil can potentiate (increase>1000-fold) the activity of atovaquone (a cytochrome bc1 or mitochondrial electron transport chain complex III inhibitor^4,5^) the atovaquone-proguanil combination, Malarone®, was developed. This alleviated concerns regarding atovaquone resistance, which developed rapidly via the spread of cytochrome bc1 mutations^6–8^. Atovaquone-proguanil is used for the treatment of uncomplicated malaria in travellers or when first-line alternative treatments are not available or effective^9^. This drug is now recommended for malaria prophylaxis^9^, with each of the drugs in this combination having been shown to have activity against *P. falciparum* liver stage parasites alone^10,11^, and in combination^12,13^.

The synergistic activity of Malarone® is widely acknowledged to be associated with interactions between atovaquone and proguanil, not between atovaquone and cycloguanil^5,14–16^. However, there is evidence that proguanil itself also has intrinsic anti-parasitic activity. This includes evidence that proguanil has weak *P. falciparum* growth inhibition activity in vitro (IC_50_ 2–71 μM; 42–72 h assays^17–19^), and thus independent of any metabolism, and that this activity is dihydrofolate reductase independent^20,21^. In vitro studies have also shown proguanil activity (IC_50_ 3.2 µM) against *P. yoelii* sporozoites infecting HepG2-CD81 human hepatoma cells. These cells have impaired P450 activity^22^ and thus limited capacity to metabolise proguanil^23^. In addition, the idea that proguanil has intrinsic activity is supported by clinical observations of efficacy in regions with high levels of cycloguanil resistance^24,25^ and in people with impaired CYP2C19 activity (i.e., poor proguanil metabolizers)^21,26^. For example, in a study on the island of Malakula, Vanuatu, high antimalarial efficacy of proguanil monotherapy was observed in patients with CYP2C19-related poor proguanil metabolizer genotypes^21^. Together, these observations suggest that, in addition to factors such as the presence or absence of pre-existing resistance of infecting parasites to atovaquone and/or cycloguanil^1,27,28^, variations in how well individuals metabolise proguanil to cycloguanil^21,26^ may have an impact on the in vivo activity of Malarone®.

The intrinsic (i.e., in absence of metabolism) in vitro activity of proguanil against asexual blood stage parasites is not completely understood, however studies have shown that parasites with impaired mitochondrial electron transport chain function are hypersensitive to proguanil^5,14,15,29^. *Plasmodium* mitochondrial DNA (6 kb) encodes three mitochondrial electron transport chain proteins: cytochrome b and cytochrome c oxidase subunits I and III^30–32^. While canonical pathways central for carbon metabolism are maintained in *Plasmodium* mitochondrion^33–36^, parasite adenosine triphosphate (ATP) requirements are primarily met by cytosolic glycolysis^35,37–40^. An important role of mitochondria in *Plasmodium* asexual intraerythrocytic parasites is the provision of pyrimidine synthesis precursors. Central to this is a mitochondrion-located, essential dihydroorotate dehydrogenase enzyme, which requires ubiquinone turnover for activity^14^. However, it is believed that *P. falciparum* parasites expressing yeast dihydroorotate dehydrogenase, whose function is not linked to parasite mitochondria, must still maintain mitochondrial membrane potential to survive^14^. Mitochondrial membrane potential, maintained by the mitochondrial electron transport chain, is thought to be required for the function of various transporters that provide substrates for essential metabolic processes in the mitochondrion, including heme biosynthesis and iron-sulphur cluster biosynthesis^14,41^. Some, but not all, tricarboxylic acid cycle enzymes also appear to be essential in asexual intraerythrocytic *Plasmodium* parasites^39^. As parasites expressing yeast dihydroorotate dehydrogenase are resistant to cytochrome bc1 inhibitors, but sensitive to proguanil in combination with a cytochrome bc1 inhibitor^5^, it has been speculated that parasites have a secondary mechanism to maintain mitochondrial membrane potential that is only essential when the mitochondrial electron transport chain is inhibited^14^. It has been hypothesised^14^ that this secondary mechanism involves ATP synthase (complex V), that may operate in reverse to hydrolyse ATP and maintain membrane potential and that proguanil inhibits this process^14^. Nevertheless, while current data suggests that proguanil sensitises parasites to atovaquone-mediated mitochondrial membrane potential collapse, such a theory does not explain the intrinsic, antiplasmodial activity of proguanil reported in multiple in vitro assays^17–19^ and supported by clinical observations^5,21,25,26^. If ATP synthase is the target of proguanil it must be essential to parasites irrespective of mitochondrial electron transport chain inhibition. Additional studies are required to explain these observations and to understand the intrinsic activity of proguanil.

**Fig. 1:**
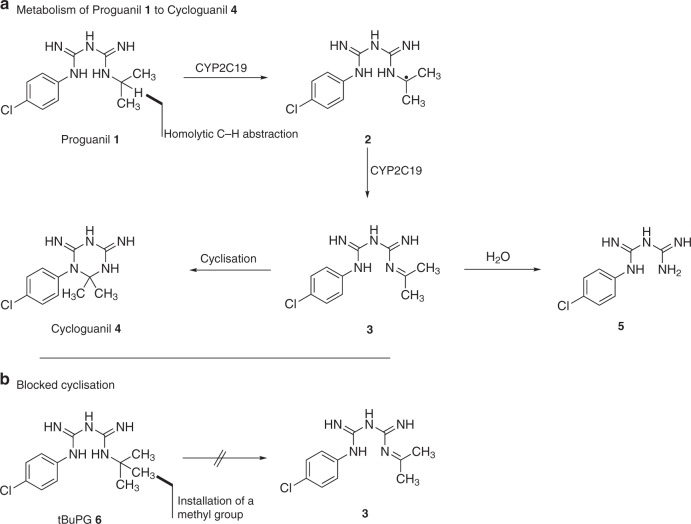
Schematic showing metabolism of proguanil to cycloguanil. **a** Proguanil (1) is metabolised to form the cyclization product cycloguanil (4) and the hydrolysis product 4-chlorophenylbiguanide (5). **b** Due to the lack of a methine proton in tBuPG (6), it is unable to undergo oxidative transformation into imine (3) and therefore cannot produce cycloguanil (4)

In this study, we explored the intrinsic^17–20^ in vitro action of proguanil against asexual intraerythrocytic stage parasites by profiling its temporal activity against different *P. falciparum* lines, demonstrating that this drug has potent, but slow-acting, activity. Furthermore, we distinguish the intrinsic anti-plasmodial activity of proguanil (1; Fig. 1) from the dihydrofolate reductase activity of cycloguanil (4; Fig. 1), by examining the activity of these compounds alongside a cyclization blocked proguanil analogue - tert-butyl proguanil (tBuPG; analogue 6; Fig. 1). tBuPG behaves similarly to proguanil, including having in vitro slow-acting activity, independent of folate, isoprenoid metabolism and pyrimidine synthesis, and synergistic activity with atovaquone and other cytochrome bc1 inhibitors. We also demonstrate antagonism between proguanil/tBuPG and cycloguanil, as well as between atovaquone and cycloguanil. Together these findings raise the possibility that a non-cyclizable proguanil analogue, similar to tBuPG, may be a better choice as a partner drug for atovaquone and other cytochrome bc1 inhibitors in vivo.

## Results

### Synthesis of a cyclization blocked proguanil analogue

**Fig. 2:**
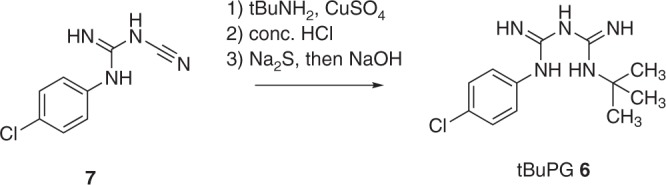
Reaction conditions for synthesis of a cyclization-blocked proguanil (tBuPG; 6)

The CYP2C19-mediated oxidation of proguanil (1; Fig. 1) involves homolytic C-H abstraction adjacent to the terminal nitrogen to give a radical (2; Fig. 1), which is oxidised (loss of a hydrogen atom) to give an imine intermediate (3; Fig. 1). This imine then either undergoes cyclisation of the adjacent nitrogen onto the imine to form cycloguanil (4; Fig. 1) or hydrolysis to form 4-chlorophenylbiguanide (5; Fig. 1). As the potent fast-action activity of proguanil (1; Fig. 1) is attributed to cycloguanil (4; Fig. 1) via the above in vivo metabolism mechanism, we synthesised the tert-butyl proguanil (tBuPG) analogue (6; Fig. 1b). In this analogue, the metabolism labile carbon-hydrogen bond is replaced with an inert carbon-carbon bond. As there is no methine proton in tBuPG (6; Fig. 1b), it is unable to undergo oxidative transformation into imine (3; Fig. 1b) and therefore cannot produce cycloguanil (4; Fig. 1). The cyclization blocked tert-butyl analogue of proguanil (6; Fig. 1b and Fig. 2) was synthesised from 3-(4-chlorophenyl)−1-cyanoguanidine (7; Fig. 2), as previously described^42^.

### Activity of proguanil and tBuPG against *P. falciparum* lines

**Fig. 3:**
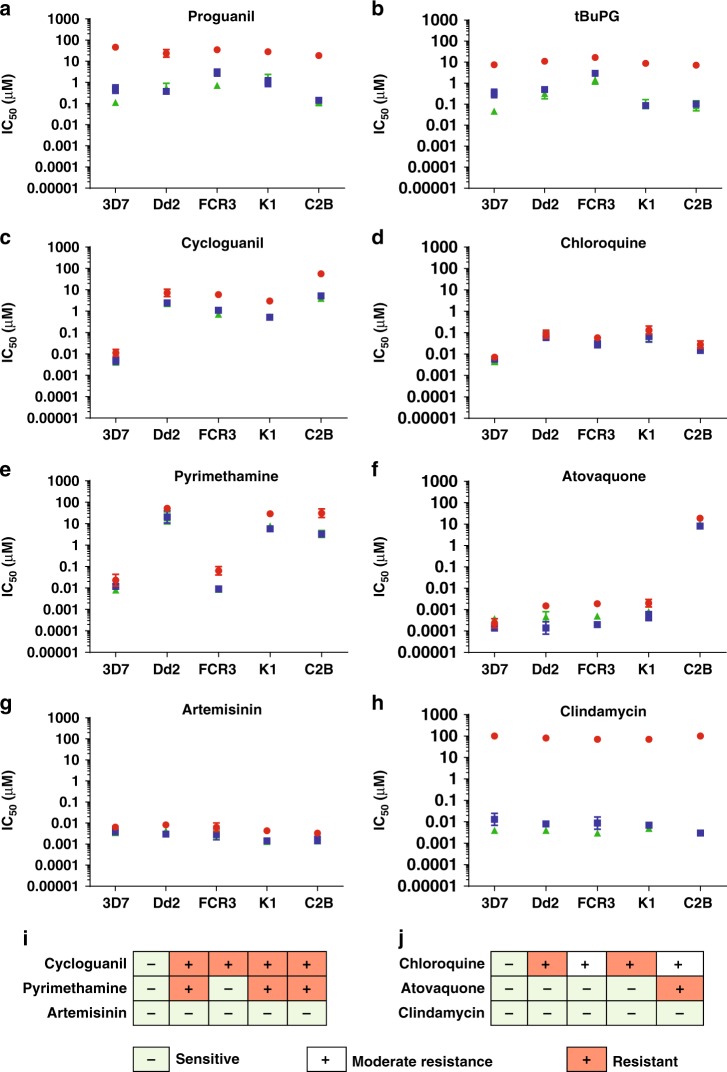
In vitro IC_50_ of proguanil, tBuPG and control antimalarials at 48 h, 72 h and 96 h*. P. falciparum* synchronous ring-stage infected erythrocytes were assayed at 48 h (red circles), 72 h (blue squares) and 96 h (green triangles) in in vitro [^3^H]-hypoxanthine uptake growth inhibition assays with different antimalarial drugs (**a**–**h**). Mean ± SD IC_50_ values (Log10 scale) are shown for at least three independent experiments, each carried out in triplicate wells. Lines were termed resistant or moderately resistant if the mean 48 h IC_50_ was > 10-fold or > 5-fold greater, respectively, than the drug-sensitive reference line 3D7 (**i** and **j**; red and white, respectively). Statistical analysis (P values) of difference between 48 h, 72, and 96 h assays for each parasite line are shown in Supplementary Table 1

**Fig. 4:**
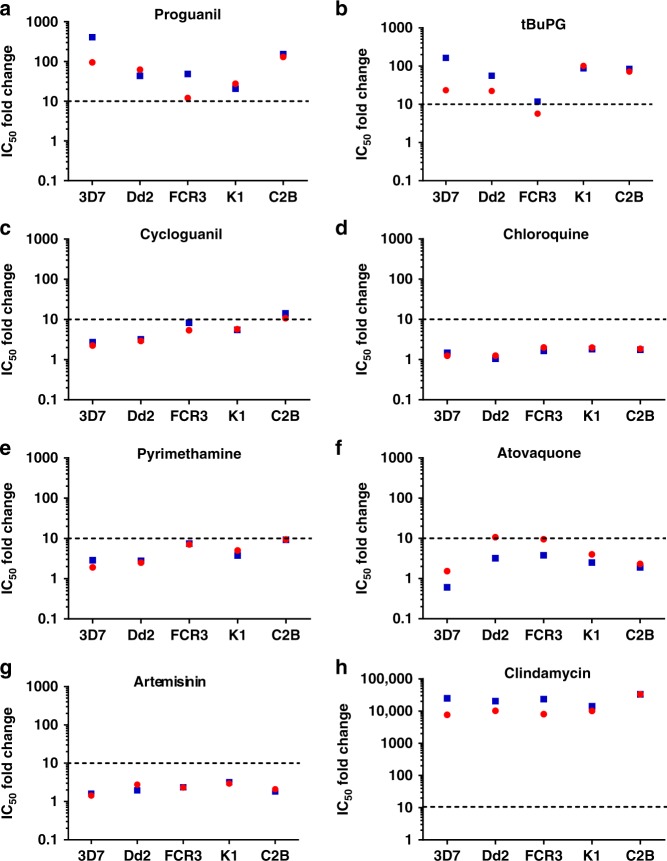
Fold-change in IC_50_ in 48 h versus 72 h or 96 h assays. *P. falciparum* synchronous ring stage infected erythrocytes were assayed in 48 h, 72 h and 96 h in in vitro [^3^H]-hypoxanthine uptake growth inhibition assays with different antimalarial drugs (**a**–**h**). Data are presented on a log-scale as fold change of mean IC_50_ values from Table1/Fig. 2 for 48 h/72 h (red) and 48 h/96 h (blue)

The in vitro activity of proguanil and tBuPG in 48 h (corresponding to one asexual developmental cycle), 72 h and 96 h (corresponding to two asexual developmental cycles) assays (Fig. 3a, b) was compared to six clinically used antimalarial drugs (cycloguanil, chloroquine, pyrimethamine, atovaquone, clindamycin, and artemisinin; Fig. 3c–h). In vitro [^3^H]-hypoxanthine uptake assays were performed using synchronous ring-stage parasites. Comparative activity was assessed using five *P. falciparum* lines (3D7, Dd2, FCR3, K1 and C2B) with differing resistance profiles to control drugs (Fig. 3i, j; Supplementary Table 1). Lines were termed resistant or moderately resistant if the mean 48 h 50% inhibitory concentration (IC_50_) was > 10-fold or > 5-fold greater, respectively, than the drug-sensitive reference line 3D7 (Fig. 3i, j, red and white, respectively). Clindamycin had  > 1000-fold better activity in 96 h (e.g., Pf3D7 IC_50_ 0.004 µM) and 72 h (e.g., Pf3D7 IC_50_ 0.013 µM) assays versus 48 h assays (e.g., Pf3D7 IC_50_ > 100 µM) (Fig. 3h; Supplementary Table 1), as expected^43^. Clindamycin is a protein synthesis inhibitor that causes a delayed death effect in *Plasmodium* with progeny inheriting a non-functional apicoplast organelle in the second cycle, following first cycle exposure^43^. The other control drugs generally showed a ten-fold or less change in IC_50_ in 48 h versus 72 h assays (Fig. 4; red circles) or 48 h versus 96 h assays (corresponding to one versus two generations of asexual blood stage intra-erythrocytic development; Fig. 4; blue squares). The IC_50_ for proguanil and tBuPG were lower at 72 h (e.g., Pf3D7 IC_50_ 0.49 µM and 0.33 µM, respectively) and 96 h (e.g., Pf3D7 IC_50_ 0.11 µM and 0.05 µM, respectively) versus 48 h (e.g., Pf3D7 IC_50_ 46.23 µM and 7.58 µM, respectively) for all *P. falciparum* lines examined (P < 0.05; Supplementary Table 1; Fig. 3a, b, respectively). The IC_50_ fold-changes for proguanil and tBuPG were generally ≥ 20x fold higher for 48 h versus 72 h or 96 h assays (Fig. 4a, b). An exception was proguanil against FCR3 in 48 h (IC_50_ 34.79 µM) versus 72 h assays (IC_50_ 2.89 µM; Fig. 4a; ~12-fold IC_50_ change) and tBuPG against FCR3 in 48 h (IC_50_ 16.76 µM) versus either 72 h (IC_50_ 2.96 µM) or 96 h (IC_50_ 1.42 µM) assays (Figure 4; ~6–12-fold IC_50_ change). None of the compounds examined (excluding clindamycin for which statistical analyses could not be carried as IC_50_s were not achieved) showed a difference in IC_50_s at 96 h versus 72 h (P > 0.01; Supplementary Table 1). Together these data indicate that proguanil and tBuPG have intrinsic slow-action activity that is not dependent on concurrent mitochondrial electron transport chain inhibition as previously hypothesised^41^.

### Proguanil and tBuPG activity is not rescued by isopentenyl pyrophosphate

**Fig. 5:**
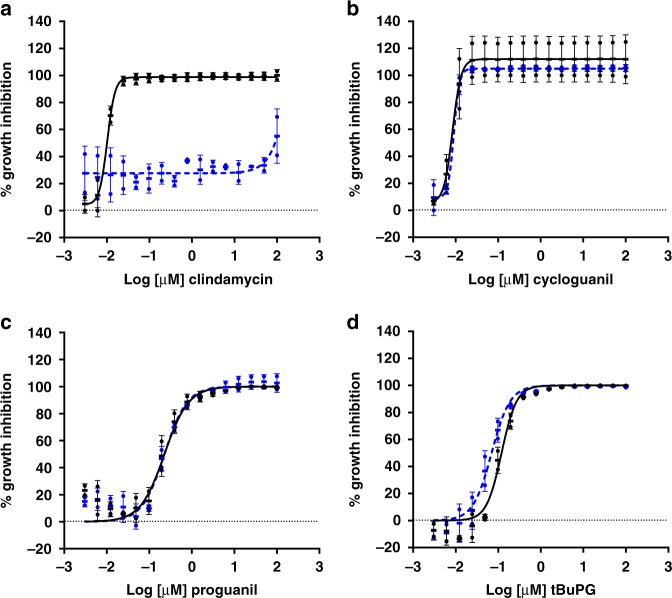
In vitro activity of proguanil and tBuPG with and without isopentenyl pyrophosphate supplementation. Synchronous ring-stage *P. falciparum* 3D7 parasitized erythrocytes were assayed using proguanil (**c**), tBuPG (**d**) or the control drugs clindamycin (**a**) and cycloguanil (**b**) over 96 h in [^3^H]-hypoxanthine uptake growth inhibition assays. Assays were carried out in RPMI 1640 parasite culture media supplemented with ( + isopentenyl pyrophosphate; blue, closed circles) or without (-isopentenyl pyrophosphate; black, closed circles) 200 µM isopentenyl pyrophosphate. In each case, data are mean ( ± SD) % inhibition of two independent experiments, each carried out in triplicate wells

Currently known slow-action antimalarial drugs including the antibiotic clindamycin, exhibit a delayed death phenotype. These compounds interfere with replication of the essential *P. falciparum* apicoplast organelle in the second cycle after exposure^43,44^. As the only essential function of the apicoplast during asexual blood stage growth is isoprenoid precursor biosynthesis, products of this pathway such as isopentenyl pyrophosphate can rescue the effects of apicoplast-associated delayed death agents in vitro^45^. To determine whether the slow-action activity observed for proguanil and tBuPG is due to apicoplast-associated delayed death, in vitro 96 h rescue assays were carried out in media with and without isopentenyl pyrophosphate supplementation. As expected, the activity of the control drug clindamycin was rescued by isopentenyl pyrophosphate (Fig. 5a). In contrast, as for the negative control compound cycloguanil (Fig. 5b), the activity of proguanil (Fig. 5c) and tBuPG (Fig. 5d) was not rescued by the addition of isopentenyl pyrophosphate. These data indicate that proguanil and tBuPG do not cause apicoplast-associated delayed death of the parasite.

### Proguanil and *t*BuPG action is not rescued by *p*-aminobenzoic acid-folic acid

**Fig. 6:**
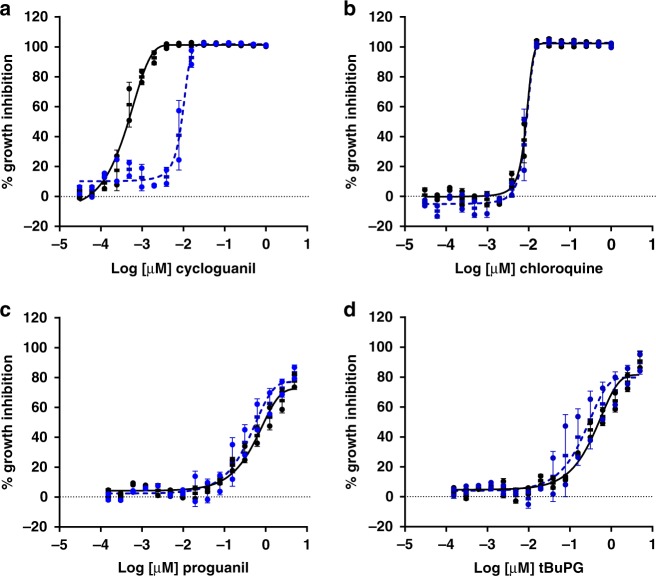
Activity of proguanil and tBuPG in low versus supplemented p-aminobenzoic acid and folic acid media. The sensitivity of synchronous ring-stage *P. falciparum* 3D7 parasitized erythrocytes to control drugs cycloguanil (**a**) and chloroquine (**b**) and test compounds proguanil (**c**) and tBuPG (**d**) was assessed in 96 h dose response [^3^H]-hypoxanthine growth inhibition assays. Assays were performed in paired plates, one containing media without supplemented *p*-aminobenzoic acid and folic acid (low *p*-aminobenzoic acid and folic acid; black solid line) and the other with supplemented *p*-aminobenzoic acid and folic acid (high *p*-aminobenzoic acid and folic acid; 1 mg /mL each; blue dashed line). Data are presented as mean ( ± SD) % inhibition of two independent experiments, each carried out in triplicate wells

To confirm that the slow-action phenotype of proguanil and tBuPG in *P. falciparum* is not related to inhibition of folate biosynthesis (mode of action of cycloguanil), rescue experiments were carried out using media supplemented with folic acid and the folate synthesis intermediate, *p*-aminobenzoic acid, versus media with low folic acid and *p*-aminobenzoic acid levels. As shown in Fig. 6, the 96 h activity of proguanil and tBuPG against *P. falciparum* 3D7 was not altered in media with low versus high levels of folic acid and *p*-aminobenzoic acid. In contrast, and as expected, the activity of cycloguanil, but not chloroquine, was rescued with folic acid and *p*-aminobenzoic acid.

### Activity appears independent of pyrimidine synthesis

**Fig. 7:**
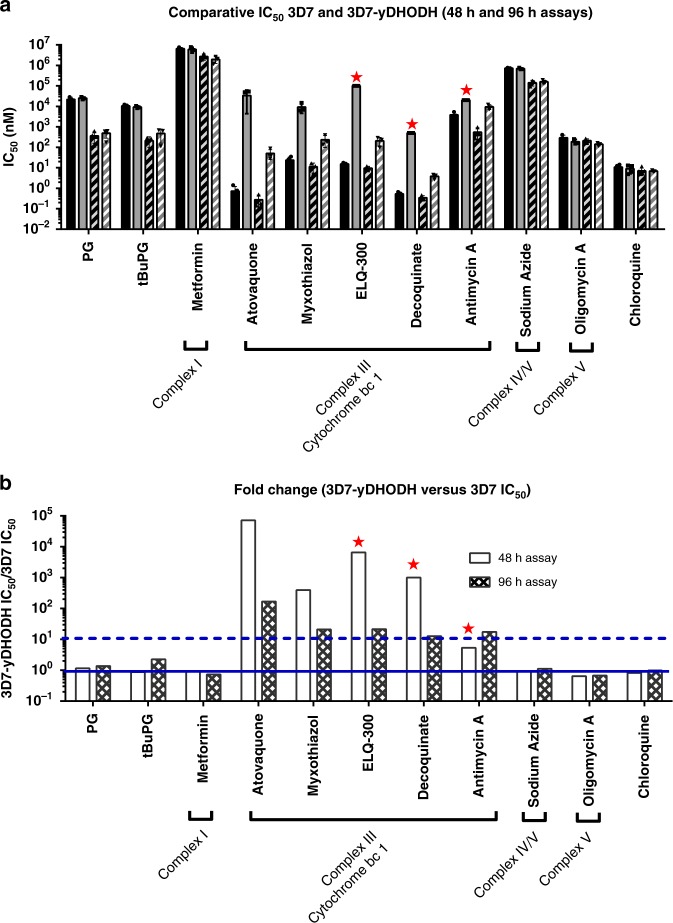
In vitro activity of compounds against *P. falciparum* 3D7 and 3D7- yeast dihydroorotate dehydrogenase parasites in 48 h and 96 h assays. **a** Compounds were tested in 48 h and 96 h assays against *P. falciparum* 3D7 and 3D7-yeast dihydroorotate dehydrogenase parasites (3D7-yDHODH), starting with synchronous ring-stage parasites. Mean IC_50_ ( ± SD) values are shown for 48 h (3D7 solid black bars; 3D7-yDHODH solid grey bars) versus 96 h (3D7 black striped bars; 3D7-yDHODH grey striped bars) assays for each line. Red star—IC_50_ not achieved (i.e., IC_50_ greater than value shown). **b** Fold change in IC_50_ of compounds against *P. falciparum* 3D7 and 3D7-yDHODH in 48 h (white bars) and 96 h (chequered bars) assays. Mean IC_50_ ( ± SD) values were used to compare activity against 3D7-yDHODH versus 3D7 for 48 h and 96 h assays. Solid and dashed horizontal lines show 1× and 10× fold change, respectively. In each case, 2–4 independent experiments were carried out, each in triplicate wells, before data were averaged and fold change calculated. Red star – IC_50_ not achieved: fold change calculated based on highest concentration tested (i.e., IC_50_ higher than this value). Selected eukaryote complex I and IV/V mitochondrial electron transport chain inhibitors (sodium azide^52,53^, oligomycin A^53^ and metformin^54^) were examined for comparison purposes

Data from previous studies show that parasites with impaired mitochondrial electron transport chain function are hypersensitive to proguanil^5,14,15,29^. A critical function of the *Plasmodium* mitochondrial electron transport chain is reoxidisation of dihydroubiquinone to ubiquinone, the electron acceptor for the dihydroorotate dehydrogenase enzyme that is critical to pyrimidine synthesis^14,46^. Here we show that proguanil and tBuPG activity appears independent of dihydroorotate dehydrogenase and pyrimidine biosynthesis by examining their activity against *P. falciparum* parasites episomally expressing yeast dihydroorotate dehydrogenase (3D7-yDHODH)^47^. As the activity of yeast dihydroorotate dehydrogenase is dependent on fumarate rather than ubiquinone, it is not linked to mitochondrial mediated ubiquinone turnover^48^. Activity against 3D7-yDHODH parasites was compared to wild type 3D7 parasites in 48 h and 96 h assays using proguanil, tBuPG and cytochrome bc1 inhibitors (atovaquone^49^, myxothiazole^49^, ELQ-300^50^, decoquinate^51^ and antimycin A^49^). As proguanil has been hypothsized to inhibit the mitochondrial electron transport chain, other eukaryote mitochondrial electron transport chain inhibitors (sodium azide^52,53^, oligomycin A^53^ and metformin^54^) were also examined for comparison purposes. However, as discussed below, the targets of these agents in *P. falciparum* are not known. The IC_50_ of proguanil was ≥ 50-fold lower for both 3D7 and 3D7-yeast dihydroorotate dehydrogenase parasites in 96 h (IC_50_ 360 nM and 490 nM, respectively) versus 48 h (IC_50_ 22.0 µM and 25.0 µM, respectively) assays (Fig. 7a, b; Supplementary Table 2; *P* < 0.01). A similar finding was seen for tBuPG for both 3D7 and 3D7-yeast dihydroorotate dehydrogenase parasites, with > 20-fold lower IC_50_ in 96 h (IC_50_ 210 nM and 470 nM, respectively) versus 48 h (IC_50_ 11.0 µM and 9.20 µM, respectively) assays (Fig. 7a, b; Supplementary Table 2; *P* < 0.01). This indicates that the slow-action activity of these compounds is retained in 3D7-yeast dihydroorotate dehydrogenase parasites. The activity of proguanil and tBuPG was also not different for 3D7 and 3D7-yeast dihydroorotate dehydrogenase parasites in 48 h or 96 h assays (3D7-yeast dihydroorotate dehydrogenase IC_50_/3D7 IC_50_ ratios of 1–2; Fig. 7b; Supplementary Table 2; *P* > 0.05). These data suggest that the slow-action activity of these compounds appears independent of pyrimidine biosynthesis, a critical pathway within *P. falciparum* mitochondria. Similar to proguanil and tBuPG, the activity of mitochondrial electron transport chain inhibitors metformin, sodium azide and oligomycin A was independent of pyrimidine synthesis, with the activity of each retained against 3D7-yeast dihydroorotate dehydrogenase parasites (3D7-yeast dihydroorotate dehydrogenase IC_50_/3D7 IC_50_ ratios of ~1–2; Fig. 7b; Supplementary Table 2; *P* > 0.05). However, in contrast to proguanil and tBuPG, metformin, sodium azide and oligomycin A did not display a slow-action phenotype (Fig. 7a; Supplementary Table 2; 3D7 96 h/48 h IC_50_ ≤ 5). As expected, the five complex III inhibitors were more potent against 3D7 than 3D7-yeast dihydroorotate dehydrogenase parasites in 48 h assays, with > 380-fold higher IC_50_s for 3D7-yeast dihydroorotate dehydrogenase versus 3D7 (excluding antimycin A, for which precise 3D7-yeast dihydroorotate dehydrogenase IC_50_ calculations were not possible; (Fig. 7a; Supplementary Table 2). This same effect was also seen for 96 h assays, with > 11-fold higher IC_50_s for 3D7-yeast dihydroorotate dehydrogenase versus 3D7 parasites (Fig. 7a; Supplementary Table 2; *P* < 0.05). Interestingly, in 96 h assays, the activity of the cytochrome bc1 inhibitors against 3D7-yeast dihydroorotate dehydrogenase parasites was >38–1000-fold greater than in 48 h assays, with the possible exception of antimycin A that was less potent than the other compounds, and for which a precise 48 h 3D7-yeast dihydroorotate dehydrogenase IC_50_ could not be calculated (Supplementary Table 2). Together these data suggest that in the absence of indirect activity due to effects on dihydroorotate dehydrogenase and pyrimidine biosynthesis, cytochrome bc1 inhibitors have a slow-action anti-plasmodial phenotype.

### In vitro combination studies

**Fig. 8:**
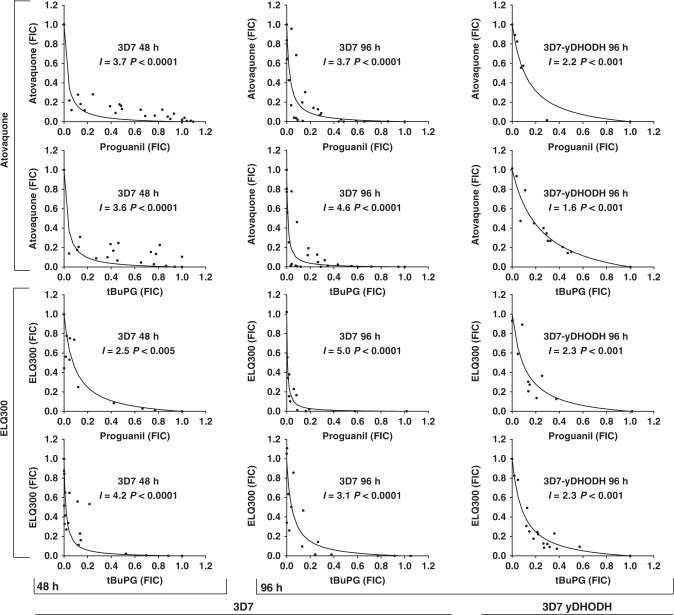
Isobolograms describing the interactions of progunail and tBuPG with cytochrome bc1 inhibitors ELQ-300 and atovaquone against *P. falciparum* 3D7 and 3D7-yeast dihydroorotate dehydrogenase (3D7-yDHODH) parasites in 48 h and 96 h assays. The interactions of antimalarial compounds was assessed using isobologram analysis. Combinations of compounds that resulted in 50% growth inhibition were determined and are presented as Fraction Inhibitory Concentrations (FICs), where a FIC = 1 represents the concentration of each compound required to inhibit parasite growth by 50% when used alone. Interaction (*I*) values were also determined^4^. Positive values of *I* indicate synergism, and negative values indicate antagonism; addition occurs when *I* equals 0. The significance of the difference of *I* from zero was assessed with Student’s t test

**Fig. 9:**
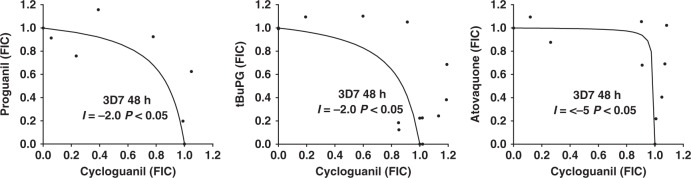
Isobolograms describing the interactions of proguanil or tBuPG with cycloguanil and cycloguanil with atovaquone against *P. falciparum* 3D7 parasites. The interactions of antimalarial compounds was assessed using isobologram analysis in 48 h assays. *I* values were calculated as previously described^4^. Positive values of *I* indicate synergism, and negative values indicate antagonism; addition occurs when* I* equals 0. The significance of the difference of *I* from zero was assessed with Student’s t test

The co-administration of compounds can result in effects that are less or greater than their predicted sum effects when used alone. These interactions can impact the activity of compounds in the clinical setting and can be assessed by examining the activity of compound combinations and then using these data to plot isobolograms. These plots indicate potential compound interactions, with concave, convex and linear isoboles being indicative of synergistic, antagonistic and additive interactions, respectively. Isobologram analyses were performed to examine the interactions of proguanil and tBuPG with cycloguanil and cyctochrome bc1 inhibitors atovaquone or ELQ-300. Assays were performed with 3D7 and 3D7-yeast dihydroorotate dehydrogenase parasites to examine compound interactions in the presence and absence of any indirect effect on pyrimidine synthesis. Proguanil and tBuPG behaved synergistically with atovaquone and ELQ-300 when assessed against 3D7 or 3D7-yeast dihydroorotate dehydrogenase parasites for 48 h or 96 h (Fig. 8), whereas combinations of cycloguanil with proguanil, tBuPG or atovaquone behaved antagonistically (Fig. 9). As 3D7-yeast dihydroorotate dehydrogenase parasites are resistant to cycloguanil, due to the presence of the human dihydrofolate reductase gene on the transfection plasmid, compound interactions with this drug against this parasite line were not assessed.

### Activity in a *Plasmodium* liver-cell infection model

**Fig. 10:**
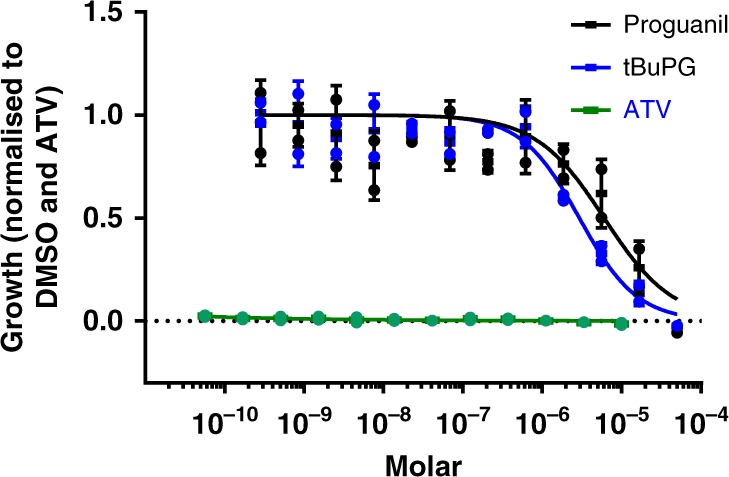
Proguanil and tBuPG show micromolar anti-*P. berghei*-Luciferase liver stage activity. Activity of proguanil and tBuPG was tested in HepG2-A16-CD81EGFP cells infected with *P. berghei*-Luciferase sporozoites. Data was normalised against atovaquone (ATV) and DMSO, and liver stage *P. berghei*-Luciferase IC_50_ values were determined using the average normalised bioluminescence intensity of 8 wells per concentration from two technical replicates of 1536- well plates, and a nonlinear variable slope four-parameter regression curve fitting model in Prism 6 (GraphPad Software Inc.). Proguanil (IC_50_ 6.02 µM) and tBuPG (IC_50_ 2.94 µM)

The effect of proguanil and tBuPG on in vitro murine malaria parasite liver stage infection^55^ was assessed using *P. berghei* luciferase-expressing sporozoites and HepG2-A16-CD81^EGFP^ cells (Fig. 10). The activity of tBuPG (IC_50_ 2.94 µM) was approximately two-fold better than that of proguanil (IC_50_ 6.02 µM). These values are consistent with the previously published activity of proguanil against *P. yoelii* infected HepG2-CD81 cells (IC_50_ 3.2 µM)^23^.

## Discussion

With almost half the world’s population at risk of malaria and a global agenda focused on eradication^56^, there is an urgent need to develop new drugs with novel modes of action for malaria prevention and treatment^57,58^. To date, the development of malaria chemoprotection drugs has lagged behind the development of treatment drugs^9,58^. However, the shift towards achieving malaria eradication has highlighted the need for new and improved chemoprotection agents^56–58^. As malaria is eliminated from countries, infection dynamics will change, with people moving from being semi-immune to non-immune. Thus, drug combinations used to prevent disease and parasite transmission (chemoprotection) described by Target Product Profile 2^58^, as opposed to those to treat malaria symptoms (treatment) described by Target Product Profile 1^58^, will be needed for vulnerable populations and during outbreaks^57^. Chemoprotection strategies should ideally include drug combinations with causal liver and asexual blood stage^58^ activity (that does not need to be fast-onset^57^), a profile that has been modelled on atovaquone-proguanil (Malarone®)^58^. Few drugs are currently available to prevent malaria, with none currently suitable for wide-scale mass drug administration in malaria-endemic areas^57,59^. Malarone® is used for daily malaria prophylaxis, but also for treatment of uncomplicated malaria in travellers or when other first-line treatments are not available or effective^9^. While doxycycline and some other antibacterial agents can be used for short-term prevention (mainly for travellers)^9^, daily dosing is prescribed and there are contraindications for infants and pregnant women, the most at-risk individuals in malaria endemic regions. Mefloquine, in contrast, can cause neurological side-effects^9^. The development of new, well-tolerated and effective chemoprotection agents is vital to the eventual eradication of malaria^57,58^.

Despite Malarone® being widely used for malaria chemoprotection, the antimalarial action of this drug combination is not completely understood. While many studies have suggested that the synergistic activity of Malarone® is mediated by atovaquone and proguanil, rather than proguanil’s active metabolite cycloguanil^16,23^, the anti-plasmodial action of proguanil has remained largely unexplored. Likewise, mechanisms to exploit the synergistic activity of atovaquone with proguanil, by preventing proguanil metabolism to cycloguanil, have not been investigated. Here we examined the possibility that a cyclization-blocked proguanil analogue may have the potential to replace proguanil through its beneficial properties as a synergistic partner drug for cytochrome bc1 inhibitors and by overcoming limitations associated with variability in how people metabolise proguanil to cycloguanil. This is important given that the activity of cyclogunail is compromised by widespread *P. falciparum* dihydrofolate reductase mutations that mediate drug resistance.

Published in vitro studies report that proguanil has weak and variable activity against asexual intraerythrocytic-stage *P. falciparum* parasites, with IC_50_s ranging from 2 to 71 μM in 42–72 h assays^17–19^. Adding to this data variability, in a high-throughput screen carried out by one of our groups several years ago, proguanil was found to have sub-µM activity in a 96 h assay (IC_50_ 0.36 µM), but poor activity in a 48 h assay (IC_50_ 2.87 μM)^60^. These data were deposited on PubChem^61,62^ as part of a larger dataset^60^, but the potential significance was not noted^60^. Indeed the dogma that proguanil has no or limited antiplasmodial activity prevails in the literature (e.g., recently reviewed in ref. ^63^), and as a result these findings remain essentially unrecognised. Here, we sought to clarify these data by investigating the intrinsic activity of proguanil and a cyclization-blocked analogue (tBuPG). Proguanil and tBuPG were first tested against five different *P. falciparum* lines in 48 h, 72 h and 96 h asexual stage, drug sensitivity assays. Our data demonstrate that proguanil and tBuPG have potent slow-action in vitro activity against *P. falciparum* parasites (Supplementary Table 1), at least an order of magnitude (10–420 fold) better than in 48 h fast-action assays. This slow-action activity is independent of folate metabolism (Fig. 6), and thus not associated with dihydrofolate reductase inhibition, cycloguanil’s primary mode of action. It is also independent of non-mevalonate isoprenoid biosynthesis (Fig. 5), the only essential function of the apicoplast organelle^45^ and the target of antibiotics with a delayed death phenotype^64^. These data suggest that slow-action activity of proguanil likely represents a different slow-action anti-plasmodial mechanism.

While proguanil’s mechanism of action is not completely understood, evidence suggests that it is linked to parasite mitochondria. In vitro combination studies have shown that proguanil lowers the concentration at which atovaquone collapses parasite mitochondrial membrane potential^14,49^. Investigations on the function of the mitochondrial ribosomal protein L13, involved in the translation of mitochondrial electron transport chain components, encoded by mitochondrial DNA, have shown that knock-down of this protein results in disruption of mitochondrial integrity, mitochondrial membrane potential collapse and hypersensitivity to proguanil^29^. The precise mechanism behind the mitochondrial membrane potential collapse mediated by proguanil is not known. However, proguanil’s poor activity in traditional fast-action assays has resulted in a hypothesis that this action is linked to an ATP synthase function that only becomes essential when the mitochondrial electron transport chain is inhibited^14^. It has been hypothesised^14^ that during mitochondrial electron transport chain inhibition, ATP synthase maintains mitochondrial membrane potential by operating in reverse and that proguanil inhibits this process^14^. This hypothesis fits well with functional observations in bovine mitochondria^54^, but does not account for reports that proguanil is active against parasites irrespective of mitochondrial electron transport chain inhibition, unless ATP synthase activity is essential to *P. falciparum* survival irrespective of mitochondrial electron transport chain inhibition. While the essentiality of this complex in *P. falciparum* remains to be determined, studies have shown ATP synthase is not essential in *P. berghei* asexual intraerythrocytic stage parasites^37,40^. It is possible that proguanil targets an alternative mechanism responsible for mitochondrial membrane potential maintenance. Alternatively, the slow-action activity of proguanil may be mediated by a mechanism different to that associated with its potentiation of mitochondrial membrane collapse.

One of the critical functions of mitochondria in malaria parasites is the re-oxidation of dihydroubiquinone to ubiquinone. Ubiquinone is the electron acceptor for several dehydrogenases including dihydroorotate dehydrogenase, a critical enzyme involved in pyrimidine synthesis^14,46^. We therefore further investigated the mode of action of proguanil by examining the effect of unlinking the pyrimidine pathway from the mitochondrial electron transport chain on proguanil and tBuPG activity. These assays involved a comparative assessment of the activity of proguanil and tBuPG with the activity of known mitochondrial electron transport chain inhibitors against wild-type *P. falciparum* parasites and parasites episomally expressing the ubiquinone-independent, fumarate-utilising, *Saccharomyces cerevisiae* (yeast) dihydroorotate dehydrogenase (*Pf*3D7-yeast dihydroorotate dehydrogenase)^47^. *P. falciparum* dihydroorotate dehydrogenase activity requires ubiquinone, however yeast dihydroorotate dehydrogenase uses fumarate, and is therefore not directly affected by cytochrome bc1 activity^14,48^. Our data demonstrate that the slow-action activity of proguanil and tBuPG is retained against *Pf*3D7-yeast dihydroorotate dehydrogenase parasites. This observation suggests that slow-action proguanil activity is independent of pyrimidine synthesis and more likely to be directly associated with mitochondrial electron potential, as previously hypothesised^14^. However, a particularly interesting observation from these data was that the activity of cytochrome bc1 inhibitors switched from comparatively fast-action when tested against 3D7 to slow-action when assessed against *Pf*3D7-yeast dihydroorotate dehydrogenase parasites. These data suggest that *Pf*3D7-yeast dihydroorotate dehydrogenase parasites remain sensitive to atovaquone, but that the activity of atovaquone changes in these parasites. While this may be explained by a separate atovaquone target, it is more likely due to mitochondrial electron transport chain inhibition given previous observations with similar parasite lines have shown that this inhibitory activity can be rescued with decylubiquinone^65^. This observation is important as it suggests the essentiality of additional ubiquinone dependent enzymes in these parasites that may warrant further investigation as drug targets. However, the strain specific nature of this observation as reported by Ke et al. requires further investigation. Interestingly, while assessments and parasites were different, Ke et al. reported yeast dihydroorotate dehydrogenase parasites derived from the 3D7 line to be resistant to slow-action atovoaquone activity^65^, we did not observe this resistance (Fig. 7 and Supplementary Table 2).

A mitochondrial mediated slow-action of atovaquone also fits well with the proposed slow-action phenotype and mechanism of action of proguanil and isobologram data that demonstrate that the interaction between atovaquone and proguanil remains synergistic against *Pf*3D7-yeast dihydroorotate dehydrogenase parasites (Fig. 8). Consistent synergy over time between atovaquone and proguanil or tBuPG against 3D7 and *Pf*3D7-yeast dihydroorotate dehydrogenase parasites also suggests that the slow-killing action of proguanil/tBuPG and atovaquone, when uncoupled from pyrimidine synthesis, are mechanistically related. While previous studies have demonstrated that proguanil and atovaquone act synergistically in vitro against wild-type^4,5^ and yeast dihydroorotate dehydrogenase-expressing parasites^14^, our study is the first, to our knowledge, to assess and compare synergy at early and late time points.

Our data on the activity of metformin, sodium azide and oligomycin A (included as eukaryotic control inhibitors of complex I, complex IV/V, and complex V, respectively) suggest that the anti-plasmodial activity of these compounds is independent of pyrimidine synthesis, but not slow-action. While these observations suggest that the slow-action mitochondrial electron transport chain inhibition phenotype may be limited to cytochrome bc1 inhibition, it is important to note that the efficacy of these classic eukaryotic mitochondrial electron transport chain inhibitors against *Plasmodium* mitochondrial electron transport chain complexes may be limited or may be complicated by other functions^66,67^. Metformin and sodium azide may have non-mitochondrial electron transport chain targets^68–70^ and have been shown to cause the generation of reactive oxygen species (ROS) following mitochondrial electron transport chain inhibition^54,71^. Thus, ROS toxicity could inhibit cells independently of mitochondrial electron transport chain dysfunction. Likewise, while oligomycin A has been shown to block the activity of ATP synthase (complex V)^72^, it has also been shown to interact with other ATPases/channels, such as Na + /K + -ATPase^73^. Interestingly, similar to ATP synthase, studies have shown that complex I (NADH:ubiquinone oxidoreductase (NDH2)) is not essential in asexual intraerythrocytic stage *P. berghei* parasites^37,40^. The essentiality of these complexes in *P. falciparum* remains to be determined.

Similar to previous studies with proguanil and atovaquone^5^, our data demonstrate that tBuPG behaves synergistically with cytochrome bc1 inhibitors (Fig. 8). Proguanil and tBuPG also interacted synergistically with ELQ-300, a cyctochrome bc1 inhibitor recently suggested as a partner drug for atovaquone^74^. ELQ-300 is an attractive combination partner for atovaquone as it binds the quinone reductase site instead of the quinol oxidase site of cytochrome bc1^75^, rendering atovaquone-resistant parasites sensitive to ELQ-300^76^. Recent data also suggest that atovaquone and ELQ-300 interact synergistically^74^. In contrast to these synergistic combinations our results show that combinations of the dihydrofolate reductase inhibitor cycloguanil with atovaquone are antagonistic (Interaction values < −5.0 in 48 h 3D7 assays; Fig. 9). While this observation has been supported by others^16^, additive activity for this combination has also been described^15^. The inconsistencies in these reports are difficult to reconcile, however, antagonistic interaction between atovaquone and WR99210, a compound with structural similarities to cycloguanil, have also been reported^15^. The mechanism of the reported antagonistic activity between atovaquone and cycloguanil remains to be determined, but may be associated with cellular entry mechanisms rather than direct mode of action. Combinations of cycloguanil with proguanil or tBuPG also behaved antagonistically in these studies (Fig. 9). These findings have potentially important ramifications for the activity of Malarone® in vivo, as they suggest that the synergistic activity of this combination may vary with proguanil metabolism and may be sub-optimal when proguanil is efficiently metabolised to cycloguanil. The rapid metabolism of proguanil to cycloguanil in a setting of high cycloguanil resistance and antagonistic activity may also impact the development of resistance to atovaquone or other cytochrome bc1 inhibitors partnered with proguanil, a factor that would be particularly problematic in instances where the partner drug has a long elimination half-life. While additional factors, including the pharmacokinetics of individual partner drugs, would need to be considered and further in vivo studies performed, these data also suggest that the highly synergistic nature of combinations of cytochrome bc1 inhibitors with proguanil can be maintained with compounds unable to be converted to cycloguanil, and that these combinations may be better than combinations with proguanil. As the cyctochrome bc1 inhibitor combination of atovaquone and ELQ-300 has recently been shown to be synergistic in *P. yoelii* infected mice^74^, it is also possible that a cyclization-blocked proguanil analogue may be a useful addition to dual cytochrome bc1 inhibitor combinations. While additional liver stage investigations, including *P. vivax* hypnozoite assays^77^, will be important to expand our findings, data showing that tBuPG (IC_50_ 2.94 µM; Fig. 10) is active against *P. yoelii* infected HepG2-CD81 cells provides additional support for a cyclization-blocked proguanil analogue and cyctochrome bc1 inhibitor combination as a chemoprevention strategy^23^.

In summary, we report that proguanil and tBuPG have potent slow-acting activity that is independent of folate metabolism, pyrimidine synthesis and isoprenoid biosynthesis. Our data support the hypothesis that these compounds function within malaria parasite mitochondria and that this activity is responsible for the synergistic activity of Malarone® but in contrast to previous hypotheses, suggest that this action is effective in the absence of concurrent mitochondrial electron transport chain inhibition. Although the slow-action of these compounds means that they may not be ideal for clinical disease use, these compounds may still be well-suited for use as prophylactics. As we have previously hypothesised^18^, our data also raise the question as to whether Malarone® is actually acting as a triple combination, dependent on where and how it is used (i.e., for chemoprotection or treatment, whether pre-existing resistance to combination components is present and whether patients are high or low proguanil metabolisers). Taking into account the pharmacokinetics of atovaquone-proguanil (proguanil t_1/2_ 12–21 h; atovaquone t_1/2_ 1–6 days^78,79^), together with the slow-action pharmacodynamics of proguanil reported here, there may be an intrinsic benefit to the use of Malarone® as a prophylactic agent. When administered for treatment Malarone® is taken for three days versus a minimum of nine days as a prophylactic^80^. The metabolism of proguanil to cycloguanil may also result in a sub-optimal combination therapy strategy given the synergy of Malarone® is driven by proguanil not its cyclized metabolite cycloguanil. Thus, an alternative, and perhaps more effective strategy, may be the incorporation of a cyclization blocked proguanil such as tBuPG or a suitable analogue. Such a strategy would also overcome variations in activity associated with differences in proguanil metabolism.

## Methods

### Parasites and culture

*P. falciparum* parasites were maintained in vitro with human O^+^ erythrocytes in RPMI 1640 (cat # R8758; Sigma) containing 10% heat inactivated human serum and 5 mg/L gentamycin at 37^o^C in 5% O_2_ and 5% CO_2_ in N_2,_ essentially as previously described^81^. The following *P. falciparum* lines were used: 3D7, Dd2, FCR3, K1 and C2B. *P. falciparum* 3D7 parasites overexpressing the yeast dihydroorotate dehydrogenase gene (3D7-yeast dihydroorotate dehydrogenase) were kindly provided by Dr Ben Dickerman, Burnet Institute, Melbourne Australia.

### Compounds

Pyrimethamine, decoquinate, myxothiazol, sodium azide, atovaquone, clindamycin hydrochloride, artemisinin, antimycin A, oligomycin A, metformin hydrochloride and chloroquine diphosphate salt were all purchased from Sigma Aldrich, USA. Cycloguanil hydrochloride was purchased from Santa Cruz Biotechnology Inc, USA. ELQ-300 was provided by the Medicines for Malaria Venture. Proguanil was purchased from Sigma Aldrich, USA or synthesised alongside cyclization blocked proguanil as described below. Isopentenyl pyrophosphate was prepared according to a literature procedure^82^ and the NMR data is in accordance with that previously reported for isopentenyl pyrophosphate^82,83^. ^1^H NMR (400 MHz, D_2_O/ND_4_OD): δ 4.86 (d, *J* = 8.8 Hz, 2 H), 4.08 (q, *J* = 6.8 Hz, *J* = 13.6 Hz, 2 H), 2.41 (t, *J* = 6.8 Hz, 2 H) and 1.78 (s, 3 H); ^13^C NMR (100 MHz, D_2_O/ND_4_OD): δ 143.9, 111.4, 64.0, 37.8 and 21.6; ^31^P NMR (161 MHz, D_2_O/ND_4_OD): δ −6.37 (d, *J* = 22.5 Hz, 1 P) and −10. 43 (d, *J* = 22.5 Hz, 1 P). All compounds were prepared as 10–20 mM stock solutions in 100% DMSO or in the case of chloroquine PBS.

Proguanil and tBuPG (; 1-(4-Chlorophenyl)−5-tert-butyldiguanide) were synthesised from 3-(4-chlorophenyl)−1-cyanoguanidine. 3-(4-chlorophenyl)−1-cyanoguanidine was prepared according to a previously published method^84^. Briefly, a solution of *p*-chloroaniline (17.0 g, 0.133 mmol) in water (63 mL) and concentrated HCl (11.1 mL) was added over 1 h to a 50 °C solution of sodium dicyanamide (23.7 g, 0.266 mmol) in water (203 mL). The reaction mixture was heated to 80 °C for 24 h. After cooling to ambient temperature, saturated NaHCO_3_ solution (150 mL) was added. After stirring for 15 min the precipitated solid was collected by filtration, washed with water and air-dried to give 3-(4-chlorophenyl)−1-cyanoguanidine as a cream-coloured solid (20.2 g, 78%). ^1^H NMR (*d*_*6*_-DMSO) 400 MHz δ 9.15 (br.s, 1 H) and 7.40–7.33 (m, 4 H); 7.07 (br.s, 2 H).

Proguanil was prepared from 3-(4-chlorophenyl)−1-cyanoguanidine according to the method of Curd and Rose (1946)^42^. 3-(4-chlorophenyl)−1-cyanoguanidine (1.0 g, 5.14 mmol) was dissolved in ethanol (7.6 mL) and water (3.0 mL) and copper sulfate were added, followed by isopropylamine (0.92 g, 15.6 mmol) and the mixture heated to reflux for 20 h. After cooling to ambient temperature, water (23 mL) was added, followed by a solution of concentrated HCl (2.6 mL) in water (15.5 mL). After stirring for 30 min a solution of sodium sulfide nonahydrate (2.06 g) in water (10 mL) was added and the mixture stirred for 30 min. The mixture was filtered to remove copper sulfide, and the filtered solid washed with hot water (50 mL). The filtrate was cooled to 10 °C and a solution of sodium hydroxide (1.16 g in water 8.3 mL) was added slowly. The precipitated solid was collected by filtration and dried. Recrystallisation from toluene gave proguanil as a colourless solid (825 mg, 63%). ^1^H NMR (*d*_6_-DMSO) 400 MHz δ 7.40 (br. s, 1 H), 7.22–7.17 (m, 2 H), 6.85–6.76 (m, 2 H), 4.89 (br. s, 2 H), 3.85–3.80 (m, 1 H) and 1.08 (d, *J* = 1.8 Hz, 6 H). HRMS (): calcd. for C_11_H_17_ClN_5_ [M]^+^ 254.1170, found 254.1167.

1-(4-Chlorophenyl)−5-tert-butyldiguanide (tBuPG) was prepared from 3-(4-chlorophenyl)−1-cyanoguanidine as follows. 1-(4-Chlorophenyl)−2-cyanoguanidine (1.0 g, 5.14 mmol) was dissolved in ethanol (7.6 mL) and water (3.0 mL) and copper sulfate (650 mg, 2.60 mmol) was added, followed by *tert*-butylamine (0.92 g, 15.6 mmol) and the mixture heated to reflux for 24 h. After cooling to ambient temperature water (23 mL) was added, followed by 2 M HCl (15.5 mL). After stirring for 30 min a solution of sodium sulfide nonahydrate (2.06 g) in water (10 mL) was added and the mixture stirred for another 30 min. The mixture was filtered to remove copper sulfide, and the solid washed with hot water (50 mL). The combined filtrate was cooled to 10 °C and a solution of sodium hydroxide (1.16 g) in water (8.3 mL) was added slowly. A cream-coloured solid precipitated and was collected by filtration. Recrystallisation from toluene gave 1-(4-chlorophenyl)−5-*tert*-butyldiguanide (436 mg, 32%) as a colourless solid. ^1^H NMR (*d*_6_-DMSO) 400 MHz δ 9.98 (br. s, 1 H), 7.76 (br. s, 1 H), 7.42–7.32 (m, 4 H), 7.22–7.00 (m, 3 H) and 1.24 (s, 9 H). HRMS: calcd. for C_12_H_19_ClN_5_ 268.1329, found 268.1327.

### In vitro *P. falciparum* growth inhibition assays

Fast-action (48 h) in vitro growth inhibitory activity of compounds was tested against synchronous early ring cultures (final 1% parasitemia and 1% haematocrit) of *P. falciparum* parasitized erythrocytes using [^3^H]-hypoxanthine incorporation (0.5 µCi/well; 96 well plates), essentially as previously described^85^. Following incubation at 37^o^C for 48 h (5% CO_2;_ 5% O_2;_ 90% N_2_) [^3^H]-hypoxanthine incorporation was determined by harvesting cultures onto glass fibre filter mats and counting using a Perkin Elmer/Wallac Trilux 1450 MicroBeta scintillation counter. Percentage inhibition of growth compared to matched vehicle controls was determined and IC_50_ values calculated using log-linear interpolation of inhibition curves^86^. Data are presented as mean ( ± SD) of at least three independent assays, each carried out in triplicate. The slow-action in vitro growth inhibitory activity of compounds was also determined using the [^3^H]-hypoxanthine incorporation against synchronous early ring cultures. However parasites were added to drug dilutions at a final parasitemia and haematocrit of 0.1% and 2% respectively. Assay plates were incubated at 37^o^C for 72 h (5% CO_2;_ 5% O_2;_ 90% N_2_) before media refreshment (100 µL) and the addition of [^3^H]-hypoxanthine (0.5 µCi/well). After a further 24 h incubation (total 96 h), [^3^H]-hypoxanthine incorporation was assessed. Growth inhibition and IC_50_ values were then determined as described for 48 h assays.

### Rescue assays

Isopentenyl pyrophosphate and folate + p-aminobenzoic acid rescue experiments were carried in 96-well plates over 96 h essentially as previously described for in vitro growth inhibition assays. However, for each comparison, two identical 96 h assays were performed simultaneously. For isopentenyl pyrophosphate rescue experiments one plate was supplemented with 200 µM isopentenyl pyrophosphate^45^ whereas the other was not. Clindamycin and cycloguanil were included as positive and negative controls respectively. For folate + p-aminobenzoic acid rescue experiments all assays were performed in custom RPMI 1640 containing no folate or p-aminobenzoic acid (Life Technologies; USA), one plate was then supplemented with folate 1 mg/mL and 1 mg/mL p-aminobenzoic acid, while the remaining plate was left without supplementation. Cycloguanil and chloroquine served as positive and negative controls respectively in these assays. In each case two independent assays were performed, each in triplicate wells.

### Isobologram assays

Antimalarial drug combinations were assessed against *P. falciparum* 3D7 or 3D7-yeast dihydroorotate dehydrogenase. Assays were performed in 96-well microtiter plates essentially as previously described^87^. However parasites were prepared at 0.1% parasitemia and 2% hematocrit for 96 h assays. Each combination was assessed at least twice in triplicate and IC_50_ values were determined by log-linear interpolation^86^. Isobolograms were constructed from normalised fractional-inhibitory concentration (FIC) values and Isoboles were fitted to define the interaction parameter, I, as previously described^4,87^. Positive values of I indicate synergism, and negative values indicate antagonism; addition occurs when I equals 0. The significance of I for each compound combination was tested using a Student’s t test^87^.

### In vitro *P. berghei*-Luciferase liver stage assays

Human hepatoma, HepG2-A16-CD81EGFP, cells were cultured at 37 °C in 5% CO_2_ in DMEM media (DMEM (Life Technology, CA) supplemented with 10% FBS and 1x Pen-Strep-Glutamine (Gibco, CA). For both *P. berghei*-Luciferase and HepG2 cytotoxicity assays, 20–26 h prior to sporozoite infection, HepG2-A16-CD81EGFP20 cells (6 × 10^5^ cells/ml in assay medium; DMEM without Phenol Red (Life Technologies, CA), 5% FBS, and 5x Pen-Strep-Glutamine (Gibco, CA)) were seeded into white solid bottom 1536-well plates (custom GNF mold ref# 789173-F, Greiner Bio-One) at 5 μL per well. Compounds (50 nL in DMSO) were transferred to wells using an Acoustic Transfer System (Biosero). Atovaquone and Puromycin were used as positive controls for *P. berghei*-Luciferase liver stage assays and HepG2 cytotoxicity assays, respectively, and tested starting at a highest concentration of 10 μM, with 1:3 serial dilutions. DMSO (0.5% DMSO final concentration) was used as negative control for both assays. *P. berghei*-ANKA-GFP-Luc-SMCON (P*. berghei*-Luciferase)^88^ sporozoites were freshly dissected from infected *Anophelese stephensi* mosquitoes received from the Insectary Core Facility at New York University (NYU). Sporozoites were then filtered twice through a 20 μm nylon net filter (Steriflip, Millipore) and 200 sporozoites/1 μL seeded into assay media. HepG2-A16-CD81EGFP cells were infected with 1 × 10^3^ sporozoites per well (5 μL) using a single tip Bottle Valve liquid handler (GNF). Plates were centrifuged at 24 °C for 3 min at 330×*g* on normal acceleration and brake settings (Eppendorf 5810 R centrifuge). HepG2-A16-CD81EGFP cells for toxicity assessment were left uninfected, with 5 µL assay media added to each well to match test plates. Plates were then incubated at 37 °C for 48 h in 5% CO_2_ with high humidity chamber. After the incubation, *P. berghei*-Luciferase exoerythrocytic form growth and HepG2-A16-CD81EGFP cell viability were assessed by a bioluminescence measurement by spinning the inverted plates at 150xg for 30 s and adding 2 μL per well of Bright-Glo (Promega) for quantification of *P. berghei*-Luciferase exoerythrocytic forms or CellTiter-Glo (Promega) (diluted 1:2 with deionized water) for quantification of HepG2-A16-CD81EGFP cell viability. Immediately after addition of the luminescence reagent, the luminescence was measured by the Envision Multilabel Reader (PerkinElmer). For IC_50_ determination, the background for the *P. berghei*-Luciferase growth inhibition was defined as average bioluminescence of the 16 wells with atovaquone (10⌠M), and the background for the HepG2 Cytotoxicity was determined as the average of the 12 wells with puromycin at single concentration (10⌠M). The baseline was defined as average bioluminescence of the 150 wells with 0.5% DMSO. IC_50_ values were determined using the average normalised bioluminescence intensity of eight wells per concentration from two technical replicates of the 1536- well plates, and a nonlinear variable slope four-parameter regression curve fitting model in Prism 6 (GraphPad Software Inc.).

### Reporting summary

Further information on experimental design is available in the Nature Research Reporting Summary linked to this article.

## Supplementary information

Supplementary Information

Description of Supplementary Data

Reporting Summary

Supplementary Data 1

## Data Availability

The authors declare that the data supporting the findings of this study are available within the paper and its supplementary information, or are available from the authors upon request. The source data underlying the plots in figures are provided in Supplementary Data 1 (Excel file).
