# Supplementary material for: Cyclization-blocked proguanil as a strategy to improve the antimalarial activity of atovaquone
Source: Commun Biol. 2019 May 3;2:166. doi: 10.1038/s42003-019-0397-3 (PMC6499835; doi:10.1038/s42003-019-0397-3)
Supplement: Supplementary file 1 — Supplementary Information [file 42003_2019_397_MOESM1_ESM.docx]

**Supplementary Table 1.** *In vitro* sensitivity of *P. falciparum* lines to anti-plasmodial compounds in 48 h, 72 h or 96 h assays.

| ***P. falciparum***  **line** | **Proguanil** | | | | | | **tBuPG** | | | | | |
| --- | --- | --- | --- | --- | --- | --- | --- | --- | --- | --- | --- | --- |
|  | **IC_50_ (µM)^a^** | | | **P value** | | | **IC_50_ (µM)^a^** | | | **P value** | | |
|  | **48h** | **72h** | **96h** | **48vs72** | **48vs96** | **72vs96** | **48h** | **72h** | **96h** | **48vs72** | **48vs96** | **72vs96** |
| **3D7** | 46.23 (±11.90) | 0.49 (±0.28) | 0.11 (±0.04) | 0.003 | 0.003 | 0.086 | 7.58 (±1.52) | 0.33 (±0.19) | 0.05 (±0.01) | 0.001 | 0.001 | 0.061 |
| **Dd2** | 23.50 (±11.98) | 0.38 (±0.38) | 0.54 (±0.37) | 0.022 | 0.023 | 0.619 | 11.08 (±2.19) | 0.50 (±0.10) | 0.32 (±0.24) | 0.001 | 0.001 | 0.302 |
| **FCR3** | 34.79 (±4.81) | 2.89 (±1.31) | 0.72 (±0.24) | 0.0004 | 0.0003 | 0.047 | 16.76 (±2.58) | 2.96 (±1.21) | 1.42 (±0.78) | 0.001 | <0.001 | 0.139 |
| **K1** | 28.19 (± 5.97) | 1.02 (±0.61) | 1.37 (±1.01 | 0.0001 | 0.0001 | 0.580 | 8.75 (±2.34) | 0.09 (±0.03) | 0.10 (±0.06) | <0.001 | <0.001 | 0.666 |
| **C2B** | 18.74 (±7.88) | 0.14 (±0.04) | 0.12 (±0.06) | 0.001 | 0.001 | 0.505 | 7.16 (±0.22) | 0.100 (±0.03) | 0.084 (±0.06) | <0.001 | <0.001 | 0.717 |
| ***P. falciparum***  **line** | **Pyrimethamine** | | | | | | **Cycloguanil** | | | | | |
|  | **IC_50_ (µM)^a^** | | | **P value** | | | **IC_50_ (µM)^a^** | | | **P value** | | |
|  | **48h** | **72h** | **96h** | **48vs72** | **48vs96** | **72vs96** | **48h** | **72h** | **96h** | **48vs72** | **48vs96** | **72vs96** |
| **3D7** | 0.023 (±0.020) | 0.012 (±0.001) | 0.008 (±0.002) | 0.391 | 0.272 | 0.045 | 0.011 (±0.005) | 0.005 (±0.002) | 0.004 (±0.001) | 0.130 | 0.073 | 0.537 |
| **Dd2** | 52.15 (±12.83) | 20.80 (±19.02) | 18.71 (±16.75) | 0.047 | 0.030 | 0.893 | 7.19 (±3.49) | 2.47 (±0.54) | 2.24 (±0.32) | 0.037 | 0.062 | 0.540 |
| **FCR3** | 0.064 (±0.036 | 0.009 (±0.003) | 0.009 (±0.002) | 0.055 | 0.054 | 0.881 | 5.97 (±1.57) | 1.10 (±0.26) | 0.72 (±0.25) | 0.006 | 0.005 | 0.144 |
| **K1** | 28.90 (±8.30) | 5.76 (±1.66) | 7.71 (±3.05) | 0.009 | 0.014 | 0.387 | 3.00 (±0.74) | 0.52 (±0.05) | 0.55 (±0.10) | 0.004 | 0.005 | 0.728 |
| **C2B** | 31.13 (±17.98) | 3.30 (±1.17) | 3.36 (±1.44) | 0.021 | 0.021 | 0.950 | 56.10 (±4.07) | 5.20 (±1.75) | 3.97 (±0.74) | <0.001 | <0.001 | 0.242 |
| ***P. falciparum***  **line** | **Chloroquine** | | | | | | **Atovaquone** | | | | | |
|  |  | **IC_50_ (µM)^a^** |  |  | **P value** |  |  | **IC_50_ (µM)^a^** |  |  | **P value** |  |
|  | **48h** | **72h** | **96h** | **48vs72** | **48vs96** | **72vs96** | **48h** | **72h** | **96h** | **48vs72** | **48vs96** | **72vs96** |
| **3D7** | 0.007 (±0.002) | 0.006 (±0.002) | 0.005 (±0.002) | 0.390 | 0.257 | 0.598 | 0.0002 (±0.0001) | 0.0002 (±0.0001) | 0.0004 (±0.0004) | 0.468 | 0.610 | 0.432 |
| **Dd2** | 0.085 (±0.046) | 0.068 (±0.036) | 0.081 (±0.044) | 0.584 | 0.898 | 0.699 | 0.0015 (±0.0004) | 0.0001 (±0.0001) | 0.0005 (±0.0003) | 0.006 | 0.028 | 0.186 |
| **FCR3** | 0.058 (±0.022) | 0.029 (±0.013) | 0.035 (±0.011) | 0.006 | 0.018 | 0.346 | 0.0019 (±0.0002) | 0.0002 (±0.0000) | 0.0005 (±0.0002) | 0.002 | 0.002 | 0.194 |
| **K1** | 0.133 (±0.071) | 0.067 (±0.055) | 0.073 (±0.064) | 0.204 | 0.315 | 0.859 | 0.002 (±0.001) | 0.0005 (±0.0003) | 0.0008 (±0.0002) | 0.037 | 0.060 | 0.163 |
| **C2B** | 0.028 (±0.014) | 0.015 (±0.006) | 0.016 (±0.006) | 0.014 | 0.029 | 0.744 | 18.98 (±4.85) | 8.18 (±3.41) | 9.99 (±3.93) | 0.017 | 0.042 | 0.513 |
| ***P. falciparum***  **line** | **Artemisinin** | | | | | | **Clindamycin** | | | | | |
|  | **IC_50_ (µM)^a^** | | | **P value** | | | **IC_50_ (µM)^a^** | | | **P value** | | |
|  | **48h** | **72h** | **96h** | **48vs72** | **48vs96** | **72vs96** | **48h** | **72h** | **96h** | **48vs72** | **48vs96** | **72vs96** |
| **3D7** | 0.007 (±0.002) | 0.005 (±0.002) | 0.004 (±0.002) | 0.384 | 0.279 | 0.820 | >100 | 0.013 (±0.012) | <0.004 | nd | nd | nd |
| **Dd2** | 0.008 (±0.001) | 0.003 (±0.001) | 0.004 (±0.001) | 0.004 | 0.005 | 0.160 | 81.57 (±19.445) | 0.008 (±0.003) | <0.004 | 0.002 | 0.011 | 0.117 |
| **FCR3** | 0.006 (±0.003) | 0.003 (±0.002) | 0.002 (±0.001) | 0.165 | 0.135 | 0.969 | 71.17 (±15.805) | 0.009 (±0.008) | <0.003 | 0.001 | nd | nd |
| **K1** | 0.004 (±0.001) | 0.001 (±0.001) | 0.001 (±0.001) | 0.003 | 0.005 | 0.745 | 71.12 (±8.156) | <0.007 | <0.005 | 0.001 | nd | nd |
| **C2B** | 0.003 (±0.001) | 0.002 (±0.001) | 0.002 (±0.001) | 0.033 | 0.150 | 0.776 | >100 | <0.003 | <0.003 | nd | nd | nd |

**^a^**The activity of all drugs was assessed against synchronous early ring cultures using [^3^H]-hypoxanthine incorporation. Data are mean (+SD) IC_50_ of at least three independent experiments, each performed in triplicate

**Supplementary Table 2.** *In vitro* activity of compounds against *P. falciparum* 3D7 and 3D7 yDHODH in 48 h and 96 h assays. The activity of all drugs was assessed against synchronous early ring cultures using [^3^H]-hypoxanthine incorporation. Data are presented as mean + SD IC_50_ of at least three independent experiments performed in triplicate.

| **Compound** | **3D7** | | | | | **3D7 yDHODH** | | | | | **yDHODH vs 3D7**  **48 h** | **yDHODH vs 3D7**  **96 h** |
| --- | --- | --- | --- | --- | --- | --- | --- | --- | --- | --- | --- | --- |
|  | **IC_50_ 48 h (M)** | | **IC_50_ 96 h (M)** | |  | **IC_50_ 48 h (M)** | | **IC_50_ 96 h (M)** | |  |  |  |
|  | **Mean** | **SD** | **Mean** | **SD** | **Δ** | **Mean** | **SD** | **Mean** | **SD** | **Δ** | **Δ** | **Δ** |
| **PG** | 2.2x10^-5^ | 6.1x10^-6^ | 3.6x10^-7^ | 2.0x10^-7^ | 61.1** | 2.5x10^-5^ | 3.2x10^-6^ | 4.9x10^-7^ | 2.1x10^-7^ | 51.0** | 1.1 | 1.4 |
| **tBuPG** | 1.1x10^-5^ | 1.8x10^-6^ | 2.1x10^-7^ | 1.1x10^-7^ | 52.4** | 9.2x10^-6^ | 1.2x10^-6^ | 4.7x10^-7^ | 3.1x10^-7^ | 19.6** | 0.8 | 2.2 |
| **Metformin** | 6.5x10^-3^ | 9.9x10^-4^ | 2.7x10^-3^ | 9.5x10^-4^ | 2.4** | 6.0x10^-3^ | 1.7x10^-3^ | 2.0x10^-3^ | 7.2 x10^-4^ | 3.0* | 0.9 | 0.7 |
| **Atovaquone** | 7.2x10^-10^ | 5.1x10^-10^ | 2.7x10^-10^ | 1.9x10^-10^ | 2.7 | 5.1x10^-5^ | 3.2x10^-6^ | 5.1x10^-8^ | 2.5x10^-8^ | 1000.0** | 72246.6** | 188.9* |
| **Myxothiazole** | 2.4x10^-8^ | 9.9x10^-9^ | 1.1x10^-8^ | 5.7x10^-9^ | 2.2 | 9.3x10^-6^ | 4.7x10^-6^ | 2.4x10^-7^ | 1.4x10^-7^ | 38.8* | 387.5** | 21.8* |
| **ELQ-300** | 1.5x10^-8^ | 2.6x10^-9^ | 9.6x10^-9^ | 2.1x10^-9^ | 1.6* | >1.0x10^-4^ | NA | 2.1x10^-7^ | 9.7x10^-8^ | >476.2 | >6666.7 | 21.9* |
| **Decoquinate** | 5.3x10^-10^ | 1.3x10^-10^ | 3.4x10^-10^ | 9.4x10^-11^ | 1.6 | >5.0x10^-7^ | NA | 3.8x10^-9^ | 9.7x10^-10^ | >131.6 | >943.4 | 11.2** |
| **Antimycin A** | 3.8x10^-6^ | 1.4x10^-6^ | 5.5x10^-7^ | 3.5x10^-7^ | 6.9* | >2.0x10^-5^ | NA | 9.5x10^-6^ | 2.5x10^-6^ | >2.1 | >5.3 | 17.3** |
| **Sodium Azide** | 7.3x10^-4^ | 3.3x10^-5^ | 1.4x10^-4^ | 4.4x10^-5^ | 5.2** | 6.9x10^-4^ | 6.8x10^-5^ | 1.6x10^-4^ | 3.3x10^-5^ | 4.3** | 0.9 | 1.1 |
| **Oligomycin A** | 2.9x10^-7^ | 9.6x10^-8^ | 2.1x10^-7^ | 5.1 x10^-8^ | 1.4 | 1.9x10^-7^ | 5.4x10^-8^ | 1.4x10^-7^ | 2.3x10^-8^ | 1.4 | 0.7 | 0.7 |
| **Chloroquine** | 1.1x10^-8^ | 3.2 x10^-9^ | 7.0 x10^-9^ | 2.7 x10^-9^ | 1.6 | 9.0x10^-9^ | 4.0x10^-9^ | 6.9x10^-9^ | 5.5x10^-10^ | 1.3 | 0.8 | 1.0 |

*P<0.05; **P<0.01
