# Supplementary material for: Cyclization-blocked proguanil as a strategy to improve the antimalarial activity of atovaquone
Source: Commun Biol. 2019 May 3;2:166. doi: 10.1038/s42003-019-0397-3 (PMC6499835; doi:10.1038/s42003-019-0397-3)
Supplement: Supplementary file 2 — Description of Supplementary Data [file 42003_2019_397_MOESM2_ESM.pdf]

Description of Additional Supplementary Files

File Name: Supplementary Data 1

Description: Tabs labelled Figure 3 and 4 and Figure 7, show 50% inhibitory concentrations (IC50) for independent experiments and mean IC50 +/- SD. Tabs labelled Figure 5 and Figure 6 show mean growth inhibition for independent experiments and mean +/- SD. Tabs labelled Figure 8 and Figure 9 show calculate fractional inhibitory concentrations (FIC) of compounds which when combined result in 50% growth inhibition. Tab labelled Figure 10 shows growth as normalized to atovaquone and DMSO vehicle as determined by bioluminescence intensity of eight wells per concentration
